# Retrograde Double-Balloon Enteroscopy Endoscopic Retrograde Pancreatography With Interventional Radiology Rendezvous of a Transplanted Pancreas

**DOI:** 10.14309/crj.0000000000001955

**Published:** 2026-01-08

**Authors:** Alexis Bayudan, Melinda Wang, Pallav Kolli, Michael Larsen

**Affiliations:** 1 San Jose Gastroenterology, San Jose, CA; 2 University of California, San Francisco, San Francisco, CA

**Keywords:** pancreatitis, pancreas transplant, ERCP, double balloon enteroscopy

## Abstract

Double-balloon enteroscopy assisted endoscopic retrograde cholangiopancreatography allows for the treatment of biliary and pancreatic diseases in patients with altered anatomy after gastrointestinal reconstruction. The percutaneous-endoscopic rendezvous technique is a well-described method that combines interventional radiology and interventional endoscopy, allowing for the cannulation of complex hepatobiliary systems through a combined endoscopic and percutaneous approach. The pancreatic transplant patient poses unique challenges in navigating the post-transplant ductal anatomy via an endoscopic approach. We describe a pancreas transplant recipient and the first report of a retrograde (scope passed through the rectum) double-balloon enteroscopy endoscopic retrograde cholangiopancreatography rendezvous to treat chronic pancreatitis.

## INTRODUCTION

Endoscopic retrograde cholangiopancreatography (ERCP) expands the ability to treat pancreatic duct pathology and is now considered first-line therapy compared with surgical management.^1^ Double-balloon enteroscopy (DBE)-ERCP is used to treat biliary and pancreatic diseases when standard ERCP scopes cannot reach the biliary tree. The pancreatic transplant patient poses unique challenges in navigating the post-transplant anatomy. The standard transplant pancreas is placed intraperitoneally and transplanted en bloc with the donor duodenum, which is anastomosed to the recipient jejunum or ileum, either directly or via an anastomosis to a Roux loop of the recipient small bowel. The percutaneous-endoscopic rendezvous technique involves interventional radiology and interventional endoscopy that allows for cannulation of complex hepatobiliary systems via access by a combined endoscopic and percutaneous approach. This technique is used as a rescue technique when standard ERCP has failed and has been described in orthotopic liver transplant patients, postgastric surgery patients, and posthepatobiliary patients.^2^

We present the first reported case of a retrograde DBE-ERCP rendezvous technique in a transplant pancreas for the treatment of chronic pancreatitis.

## CASE REPORT

**Figure 1.**
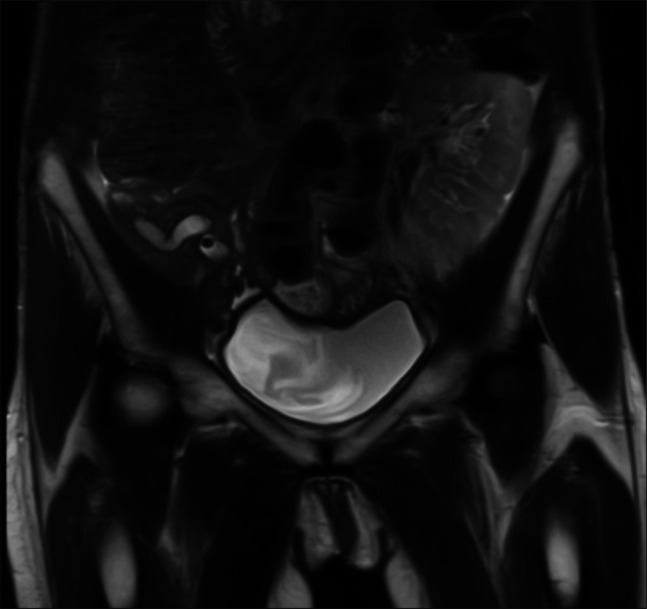
MRCP coronal T2-weighted sequence demonstrates the right lower quadrant transplant allograft and dilation of the main pancreatic duct to 1.0 cm containing nonobstructing stones. MRCP, magnetic resonance cholangiopancreatography.

A 67-year-old woman with diabetes mellitus type I status post simultaneous pancreas and kidney transplant with exocrine drainage to the bladder with subsequent enteric conversion due to chronic urethritis, presents with recurrent pancreatitis of her transplanted pancreas. She also had a small bowel obstruction with small bowel resection with jejunojejunal anastomosis near the transplant pancreas duodenojejunal anastomosis. She was hospitalized with symptoms of pain and found to have elevated amylase 920 U/L and lipase 819 U/L. Ultrasound revealed dilation of the main pancreatic duct, most pronounced at the head, measuring up to 9 mm with 2 stones in the ampulla. Magnetic resonance cholangiopancreatography (Figure 1) demonstrates markedly dilated main pancreatic duct and secondary uncinate duct, tapering smoothly to the ampulla, containing nonobstructing stones.

Given improved pain and laboratory test results, she was discharged and subsequently underwent an attempt at an outpatient retrograde DBE/ERCP. Retrograde DBE demonstrated 3 lumens at the jejunojejunal anastomosis. One limb was a blind-ending pouch containing the transplanted duodenum and the major papilla. After exhaustive attempts, including using contrast with fluoroscopy, administration of secretin, and discussion with the transplant team intraprocedurally, the ampulla could not be identified. The procedure was terminated with a plan for rendezvous ERCP with interventional radiology.

**Figure 2.**
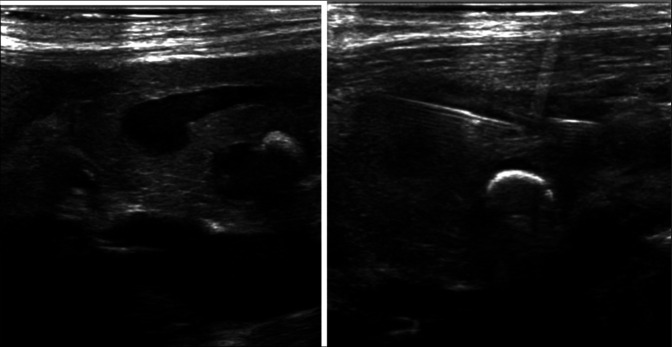
The dilated pancreatic duct was accessed via a direct percutaneous puncture under ultrasound guidance. A large shadowing stone can be seen in the dilated uncinate duct.

**Figure 3.**
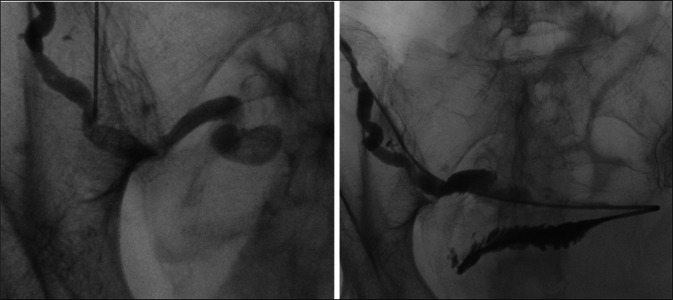
Contrast injection through percutaneous access demonstrates a dilated pancreatic duct with severe ampullary narrowing. The obstruction was crossed with a 0.035-inch Advantage Glidewire and contrast injection confirmed the location in the small bowel.

She subsequently returned for the rendezvous procedure. With the patient in a supine position under general anesthesia, percutaneous access to the dilated main pancreatic duct in the head of the transplant pancreas was obtained using a 21-gauge needle under direct ultrasound visualization (Figure 2). A 0.018-inch Cope wire was advanced into the pancreatic duct but did not pass spontaneously across the ampulla. Multiple attempts to pass a wire into the small bowel were unsuccessful, and an attempt to use a Neff conversion set to upsize access resulted in loss of access. Next, a new access site in the transplanted pancreas body was identified and accessed, again using a 21-gauge needle under direct ultrasound guidance. A 0.018-inch Cope wire was advanced into the pancreatic duct and used to upsize to a 5 French sheath. Using a combination of a 5-French Kumpe catheter and 0.035-inch Glidewire Advantage, the ampullary obstruction was crossed, and after fluoroscopic confirmation of location, an Amplatz wire was advanced into the bowel, and the patient was positioned on her left side for retrograde DBE (Figure 3).

**Figure 4.**
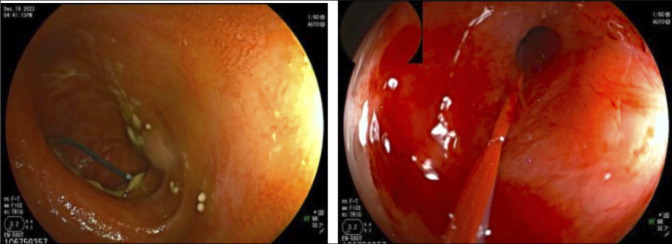
Endoscopic view of the Cope wire exiting ampulla with subsequent pancreatic duct orifice cannulated with the metro wire post balloon dilation.

**Figure 5.**
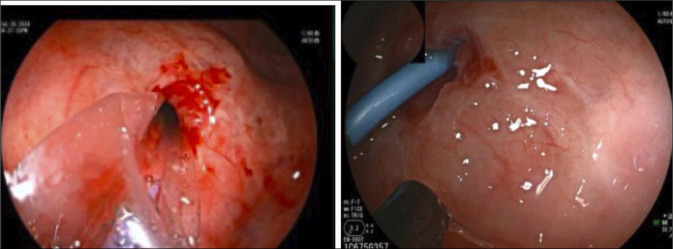
Balloon dilation and stent placement into the pancreatic duct orifice.

The DBE then passed through the lower gastrointestinal tract with identification of the jejunojejunal anastomosis and evidence of 3 lumens, with 1 limb containing the percutaneous sheath and wire exiting the pancreatic ampulla. The ampulla appeared severely stenosed. Using a cannulatome, a 0.035-inch straight wire was passed into the main pancreatic duct alongside the percutaneous sheath (Figure 4). Dilation of the papilla to a maximum balloon diameter of 8 mm was performed. A 7 French by 5 cm double pigtail stent was placed into the pancreatic duct (Figure 5).

**Figure 6.**
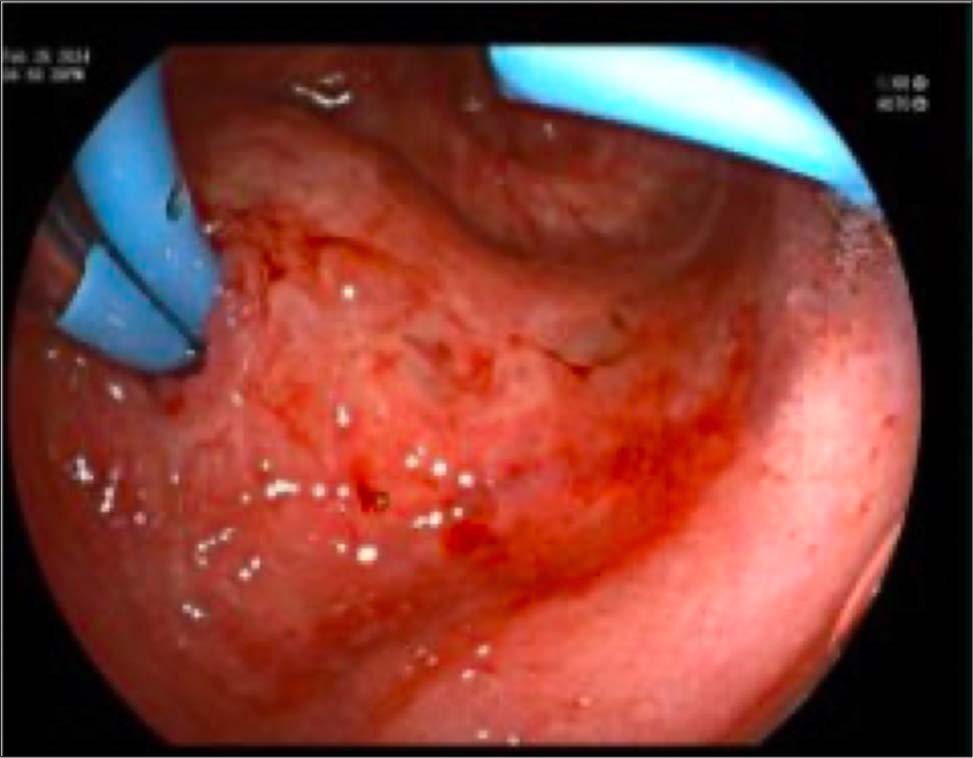
Repeat procedure 3 months later with patent orifice and stent exchange with 2 plastic stents.

Following the procedure, abdominal pain improved and was rated 0/10 at discharge. At clinic follow-up 1 month postprocedure, the patient reported 90% improvement in abdominal pain. Ultrasound imaging performed demonstrated unchanged positioning of the pancreatic duct stent with improvement in pancreatic duct dilation to 0.4 cm. At 3 months postprocedure, lipase levels decreased to 65 U/L, and repeat ERCP demonstrated a stent in place and improvement in pancreatic duct orifice stricture. The previous stent was removed, the orifice was dilated to 7 mm using the CRE wire-guided balloon, and 2 new 7 French by 5 cm stents were placed (Figure 6). At 12.5 months from her initial procedure and with 6 months of being stent-free, she continues to do well.

## DISCUSSION

This case highlights the first instance of a combined retrograde DBE-assisted ERCP rendezvous technique in a transplant pancreas. A thorough review of surgical history and previous imaging is crucial for procedural planning and to safely access the pancreatic duct via both endoscopic and percutaneous techniques. When the pancreas transplant is anastomosed to the distal jejunum or ileum, retrograde DBE is necessary to gain access to the ampulla. Using DBE-ERCP, therapeutic interventions such as pancreatic duct dilation, stone removal, and stent placement can be performed. The ampulla was not identified by retrograde DBE-ERCP alone, and a rendezvous technique was implemented.

Percutaneous access with a small gauge needle (21-gauge or smaller) minimizes the risk of procedure-induced pancreatitis and damage to surrounding structures, including bowel. Percutaneous access into the tail of the transplanted pancreas allows access along the longest length of the duct as well as the ability to exchange for more stable access, such as a sheath. Once the ampulla has been crossed, the endoscopic rendezvous technique can be used to either cannulate the ampulla directly or establish through-and-through access.

This case demonstrates that pancreatic endotherapy for pancreas transplants can be performed with balloon-assisted ERCP. This case contributes to the growing body of literature regarding advanced access techniques of the pancreas to allow for therapeutic procedures. Collaborative interventions such as the percutaneous-endoscopic ERCP rendezvous technique may be considered in complex cases with nonnative anatomy for optimal patient outcomes.

## DISCLOSURES

Author contributions: A. Bayudan: Drafting of the manuscript. M. Wang: Drafting of the manuscript. P. Kolli: Drafting of the manuscript. M. Larsen: Drafting of the manuscript, final approval, and is the article guarantor.

Financial disclosure: Alexis Bayudan: Consultant for Olympus. Melinda Wang: None. Pallav Kolli: Consultant for Becton, Dickinson and Company. Michael Larsen: None.

Informed consent was obtained for this case report.

## ABBREVIATIONS:

DBE: double-balloon enterography
ERCP: endoscopic retrograde cholangiopancreatography
MRCP: magnetic resonance cholangiopancreatography
